# Marked Independent Relationship between Circulating Interleukin-6 Concentrations and Endothelial Activation in Rheumatoid Arthritis

**DOI:** 10.1155/2013/510243

**Published:** 2013-12-21

**Authors:** Patrick H. Dessein, Ahmed Solomon, Angela J. Woodiwiss, Gavin R. Norton, Linda Tsang, Miguel A. Gonzalez-Gay

**Affiliations:** ^1^Cardiovascular Pathophysiology and Genomics Research Unit, School of Physiology, Faculty of Health Sciences, University of the Witwatersrand, P.O. Box 1012 Johannesburg, Melville 2109, South Africa; ^2^Department of Rheumatology, Charlotte Maxeke Johannesburg Academic Hospital, Faculty of Health Sciences, University of the Witwatersrand, South Africa; ^3^Milpark Hospital, Johannesburg, South Africa; ^4^Department of Rheumatology, Hospital Universitario Marques de Valdecilla, IFIMAV, Avenida de Valdecilla s/n, 39008 Santander, Spain

## Abstract

We examined the potential impact of conventional compared with nonconventional cardiovascular risk factors including interleukin-6 levels on endothelial activation in RA. Circulating soluble E-selectin, vascular cell adhesion molecule-1, intercellular adhesion molecule-1, and monocyte chemoattractant protein-1 concentrations were measured in 217 African patients (112 black and 105 white) with RA. In comprehensive confounder adjusted mixed regression models, 5 conventional and 4 nonconventional cardiovascular risk factors were associated (*P* = 0.05 to <0.0001) with endothelial activation. Interleukin-6 was the only risk factor related to each endothelial activation molecule and independently contributed by 18% and significantly more than other risk factors to the variation in overall endothelial activation as estimated by an SD (z) score of endothelial activation molecule concentrations. The independent interleukin-6-overall endothelial activation relationships were reproduced in various subgroups. Interleukin-6 concentrations relate consistently, markedly, and to a larger extent than other cardiovascular risk factors to endothelial activation in RA. Assessment of interleukin-6 concentrations may enhance cardiovascular risk stratification in RA.

## 1. Introduction

Atherogenesis is increasingly recognized as a dynamic and inflammatory process [1–3] and is initiated by activation of endothelial cells upon exposure to cardiovascular risk factors. This results in enhanced expression of soluble forms of selectin, vascular cell adhesion molecule-1 (VCAM-1), and intercellular adhesion molecule-1 (ICAM-1). These molecules promote adherence of monocytes to the endothelium. Once adherent, monocytes penetrate the endothelial lining and enter the intima of the arterial wall by diapedesis between endothelial cells. This monocyte transmigration is largely effectuated by monocyte chemoattractant protein-1 (MCP-1) that interacts with the monocyte receptor CCR2. Indeed, MCP-1 plays a unique and crucial role in the initiation of atherosclerosis [4].

Circulating concentrations of molecules that mediate endothelial activation associate with atherosclerotic cardiovascular disease in the general population [5–7]. Patients with RA experience enhanced endothelial activation that associates with endothelial dysfunction as assessed by endothelial dependent flow-mediated dilatation and carotid atherosclerosis [8–11]. These observations support the contribution of systemic inflammation to the now well-documented excess cardiovascular disease burden in patients with RA [12–15]. Conventional cardiovascular risk factors and disease characteristics relate independently of one another to both atherosclerosis and cardiovascular event rates in RA [16–18]. However, these cardiovascular outcomes result from the cumulative effects of cardiovascular risk factor exposure accrued during the entire lifespan [9, 10]. By contrast, endothelial activation relates more closely to the effects of cardiovascular risk factors present at the time of evaluation and can be rapidly reversed by treatment with cardioprotective drugs [9–11] and, in RA, by effective suppression of cytokine production or cytokine blockade [19, 20]. Enhanced endothelial activation precedes increased atherosclerosis in RA [10]. Evaluation of endothelial activation further provides insight in atherogenic mechanisms that can be disease [11, 21] and population specific [22]. This has implications in individualized cardiovascular risk assessment [22].

Available data on potential determinants of endothelial activation in RA is limited [9]. Interleukin-6 (IL-6) may be particularly important in this context [9, 23–26]. This cytokine is not only a major circulating cytokine [9] that relates most closely to clinical disease activity in RA [26] but also promotes synovitis by inducing neovascularization, infiltration of inflammatory cells, and synovial hyperplasia [23]. Indeed, IL-6 blockade is highly effective in controlling RA activity [23, 24]. Overproduction of IL-6 further increases very-low-density lipoprotein receptor expression and may thereby mediate disease activity-related reduced lipid concentrations in RA [25]. In this study, we systematically examined the potential impact of conventional compared with non-conventional cardiovascular risk factors including IL-6 on endothelial activation in a relatively large RA cohort.

## 2. Materials and Methods

### 2.1. Patients

The present investigation was conducted according to the principles outlined in the Helsinki declaration. The Committee for Research on Human Subjects of the University of Witwatersrand approved the protocol (approval number M06-07-33). Patients gave informed written consent. The present study design has previously been described [27–29]. Briefly, 217 African patients (112 black and 105 white) that met the 1987 American College of Rheumatology criteria [30] were enrolled at the Charlotte Maxeke Johannesburg Academic Hospital and Milpark Hospital. All invited patients agreed to participate. Cardiovascular medication use was recorded. Disease modifying agents for rheumatic disease (DMARD) were employed in each patient at the time of the study. This included the use of biological agents (tumor necrosis alpha blockade (*n* = 8) and rituximab (*n* = 1)). Amongst nonbiological DMARD, methotrexate, chloroquine, leflunomide, sulphasalazine, azathioprine, tetracycline, cyclophosphamide, and penicillamine were employed by 84.8, 66.8, 29.5, 20.3, 14.8, 10.6, 3.7, and 2.8% of patients, respectively. In 6 patients on prednisone, the doses used were 2.5 (*n* = 3), 5 (*n* = 1), 10 (*n* = 1), and 12 (*n* = 1) mg daily. Data were missing in fewer than 5% of any of the recorded characteristics.

### 2.2. Clinical Characteristics

We recorded demographic features and smoking status (current and ever). Height, weight and waist and hip circumference were measured by standard approaches. We calculated the body mass index (BMI) as a measure of overall adiposity and abdominal adiposity and fat distribution were estimated by waist circumference and waist-hip ratio, respectively [27]. Hypertension was defined as an average systolic blood pressure ≥140 or/and diastolic blood pressure ≥90 mmHg or/and current use of antihypertensive agents. Fasting blood samples were taken between 8:00 and 10:00 am. Standard laboratory tests of renal function, lipids and glucose were performed. Dyslipidemia was diagnosed when the atherogenic index, that is, the cholesterol-HDL cholesterol ratio, was >4 and proatherogenic non-HDL cholesterol concentrations were calculated by subtracting HDL cholesterol from total cholesterol concentrations [31, 32]. Diabetes was identified as the use of glucose lowering agents or a fasting plasma glucose ≥7 mmol/L. We calculated the estimated glomerular infiltration rate (EGFR) using the modification of diet in renal disease (MDRD) equation [33]. Evaluated RA characteristics included disease duration, rheumatoid factor status, and the number of deformed joints as an indicator of disease severity or joint damage [16], the disease activity score in 28 joints (DAS28) [34], and the erythrocyte sedimentation rate (ESR) and prednisone use. C-reactive protein (CRP) concentrations were evaluated by immunoturbidimetric methods. In 132 patients, this was done on the DxC/LX analyzer (Beckman Coulter Inc, Brea, USA) with a lower detection limit of 1 mg/L and the inter- and intra-assay coefficients of variation of 2.5 and 5.0%, respectively; in the remaining 85 patients, CRP concentrations were quantified on the AU analyzer (Olympus, Essex, UK) with a lower detection limit of 0.05 mg/L and inter- and intra-assay coefficients of variation of 1.3 and 0.4 mg/L, respectively. In the general population, a CRP concentration > 1 mg/L reportedly predicts increased incident cardiovascular disease [35, 36]. Seventy-four blood samples from subjects that did not participate in the present study were tested on both the AU and DxL/LX systems and the Spearman correlation coefficient between CRP values was 0.994. We measured IL-6 concentrations using a solid-phase sandwich enzyme-linked immunosorbant assay (ELISA) (Quantikine HS, R&D Systems, Inc., Minneapolis, MN, USA). The lower detection limit ranged from 0.016 to 0.110 pg/mL and the inter- and intra-assay coefficients of variation were 7.8 and 7.4%, respectively.

### 2.3. Endothelial Activation Molecules

We measured circulating soluble VCAM-1, ICAM-1, E-selectin and MCP-1 concentrations using a solid-phase sandwich ELISA (Quantikine HS, R&D Systems, Inc., Minneapolis, MN, USA). Their lower detection limits were 0.6 ng/mL, 0.096 ng/mL, 0.009 ng/mL and 5.0 pg/mL, respectively; their inter- and intra-assay coefficients of variation were 7.0 and 3.1, 5.5, and 4.6, 7.9, and 5.8, and 5.7 and 5.8%, respectively.

### 2.4. Resistin Concentrations

Patients in the present study also had their circulating resistin concentrations determined upon participation in another investigation [37] and employing a solid phase sandwich ELISA (Quantikine HS, R&D Systems, Inc., Minneapolis, MN, USA). The lower detection limit was 0.026 ng/mL and the inter- and intra-assay coefficients of variation were 7.8 and 7.4%, respectively.

### 2.5. Data Management and Analysis

Dichotomous variables are expressed as proportions or percentages and continuous variables as mean (SD) and median (interquartile range) when normally and nonnormally distributed, respectively. Nonnormally distributed characteristics were logarithmically transformed prior to including them in mixed regression models. An endothelial activation score was used to provide a summary measure of endothelial activation and was calculated from SD (*z*) scores as follows: [*z* (VCAM-1) + *z* (ICAM-1) + *z* (E-selectin) + *z* (MCP-1)]. This score was recently successfully employed in documenting a potential independent role of resistin, a proinflammatory adipokine, in cardiovascular risk and its stratification in RA [37].

The relationships of clinical characteristics including conventional cardiovascular risk factors and RA characteristics with endothelial activation molecule concentrations and the endothelial activation score were first assessed in age, sex and cardiovascular drug use adjusted multivariable models and, subsequently, in models that included age and sex and other conventional cardiovascular risk factors as well as RA characteristics. Our previous investigations showed consistent disparities in cardiovascular risk factor-cardiovascular disease associations in black compared to white African patients with RA [27–29]. Therefore, we systematically determined the impact of population origin or, in the present context, also ethnic grouping (EG) on the cardiovascular risk factor-endothelial activation relationships by the addition of interaction terms (EG *x* variable of interest) and their individual terms to the models and in stratified analysis, that is, in black and white patients separately [27–29].

To further ensure that the strongest cardiovascular risk factor-endothelial activation associations identified in the analysis could not be attributed to any specific characteristic including EG of the RA cohort studied, we performed sensitivity analysis separately in black and white patients, women, men, participants lesser/equal and >55 years, nonobese and obese patients, normotensives and hypertensives, participants with a cholesterol-HDL cholesterol ratio of less/equal and >4, a disease duration of less/equal and >10 years, rheumatoid factor positive and negative, no/mild and moderate/high disease activity [29], and less/equal and >6 deformed joints (median number of deformed joints in all patients was 6). The endothelial activation score was the dependent characteristic in these models. Sensitivity analysis was not performed in diabetic and nondiabetic patients in view of the small number of patients with this comorbidity.

A *P* value of ≤ 0.05 was considered statistically significant. Statistical computations were made using the GB Stat program (Dynamic Microsystems, Inc., Silverspring, Maryland, USA).

## 3. Results

### 3.1. Clinical Characteristics and Endothelial Activation Molecule and Resistin Concentrations in Patients with RA

As given in Table 1, most conventional and non-conventional risk factors as well as endothelial activation molecule concentrations differed by ethnic grouping.

### 3.2. Age, Sex, and Cardiovascular Drug Adjusted Relationships of Modifiable Cardiovascular Risk Factors and Disease Characteristics with Endothelial Activation in Patients with RA

Age and sex were not associated with any of the evaluated endothelial activation molecule concentrations (data not shown).

In age, sex, and cardiovascular drug use adjusted analysis in all patients, IL-6 concentrations were associated with those of each endothelial activation molecule (partial *R* = 0.330 (*P* < 0.0001), 0.244 (*P* = 0.0004), 0.155 (*P* = 0.02), and 0.265 (*P* = 0.0001) for MCP-1, VCAM-1, ICAM-1, and E-selectin) as well as the endothelial activation score (partial *R* = 0.479 (*P* < 0.0001)). Population grouping impacted on the relationship between IL-6 and MCP-1 concentrations (interaction *P* = 0.0005). Nevertheless, in stratified analysis, IL-6 concentrations associated with those of MCP-1 in both black and white patients with RA (partial *R* = 0.489 (*P* < 0.0001) and 0.210 (*P* = 0.038), resp.).

In all patients and irrespective of EG (interaction *P* > 0.05), waist circumference, number of deformed joints, and ESR were additionally associated with VCAM-1 concentrations (partial *R* = − 0.136 (*P* = 0.048), 0.186 (*P* = 0.007), and 0.149 (*P* = 0.034) resp.), ever smoking with ICAM-1 concentrations (partial *R* = 0.142 (*P* = 0.035)), hypertension, diastolic blood pressure, ESR and glucose concentrations with those of E-selection (partial *R* = 0.176 (*P* = 0.01), 0.144 (*P* = 0.037), 0.227 (*P* = 0.001) and 0.147 (*P* = 0.036) resp.) and EGFR and prednisone with the endothelial activation score (partial *R* = − 0.179 (*P* = 0.01) and 0.157 (*P* = 0.02), resp.).

Population grouping impacted (interaction *P* < 0.05) on the relationships of disease duration and EGFR with MCP-1 concentrations, ESR with VCAM-1 and ICAM-1 concentrations and the endothelial activation score, and LDL cholesterol concentrations with those of ICAM-1. Subsequent stratified analysis revealed that, in black patients, LDL cholesterol was associated with ICAM-1 concentrations (partial *R* = − 0.219 (*P* = 0.03)) and ESR with VCAM-1 concentrations and the endothelial activation score (partial *R* = 0.208 (*P* = 0.038) and 0.273 (*P* = 0.004), resp.); in white patients, disease duration was associated with MCP-1 concentrations and the endothelial activation score (partial *R* = 0.275 (*P* = 0.006) and 0.241 (*P* = 0.017), resp.) and the EGFR with MCP-1 concentrations (partial *R* = −0.233 (*P* = 0.02)).

### 3.3. Independent Relationships between Modifiable Cardiovascular Risk Factors and Endothelial Activation

The associations between modifiable cardiovascular risk factors and endothelial activation in models adjusted for age and sex as well as other cardiovascular risk factors including conventional ones (BMI, hypertension, a cholesterol-HDL cholesterol ratio >4, use of lipid lowering agents, smoking and diabetes) and RA characteristics (disease severity (number of deformed joints), DAS28, and prednisone use) as potential confounding or explanatory variables are shown in Table 2. Additionally, in black patients, LDL cholesterol concentrations were associated with those of ICAM-1 (partial *R* = − 0.211 (*P* = 0.04)) and ESR with VCAM-1 concentrations and the endothelial activation score (partial *R* = 0.199 (*P* = 0.05) and 0.283 (*P* = 0.005)) in comprehensively adjusted analysis; in white patients, disease duration associated with MCP-1 concentrations and the endothelial activation score (partial *R* = 0.279 (*P* = 0.008) and 0.243 (*P* = 0.02)) and EGFR with MCP-1 concentrations (partial *R* = − 0.231 (*P* = 0.03)).

In a separate model in all patients and in which both IL-6 concentrations and ESR were entered, each systemic inflammation marker independently associated with E-selectin concentrations (partial *R* = 0.229 (*P* = 0.002) and 0.190 (*P* = 0.009) resp.).

**Figure 1:**
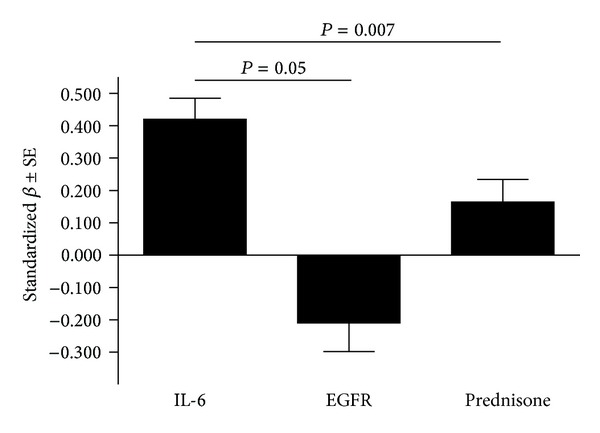
Comparison of the magnitude of the effect of interleukin-6 concentrations versus those of the estimated glomerular infiltration rate and prednisone use on the endothelial activation score in models in Table 2.

As derived from the partial *R* ^2^ *s* in the models with the endothelial activation score as the dependent variable that produced the results in Table 2, IL-6 concentrations independently contributed 17.9% to the variation in the endothelial activation score, whereas the corresponding values for EGFR and prednisone were 4.2 and 2.7% respectively. A comparison of the magnitude of the effect of interleukin-6 concentrations versus those of EGFR and prednisone use on the endothelial activation score in models in Table 2, is given in Figure 1.

In the model shown in Table 3, we entered those risk factors, except for diastolic blood pressure and disease duration (in view of their collinearity with hypertension and number of deformed joints resp.), that were independently associated with endothelial activation in the previous analysis together with other cardiovascular risk factors and with the endothelial activation score as the dependent variable. Demographic characteristics were not associated with endothelial activation. Amongst the conventional risk factors, only the EGFR was associated with the endothelial activation score. By contrast, amongst the non-conventional cardiovascular risk factors, IL-6 concentrations, ESR, and current prednisone use remained associated with endothelial activation.

Table 4 gives the results obtained in sensitivity analysis. Independent of the risk factors adjusted as in Table 2, IL-6 concentrations remained significantly associated with the endothelial activation score in black and white patients, women, men, participants lesser/equal or >55 years, nonobese and obese patients, normotensives and hypertensives, participants with a cholesterol-HDL cholesterol ratio of less/equal and >4, a disease duration of less/equal and >10 years, rheumatoid factor positive and negative (borderline with *P* = 0.08), no/mild and moderate/high disease activity [34], and less/equal and >6 deformed joints. The association of the IL-6 concentrations with the endothelial activation score was also significant and approached significance in rheumatoid positive and rheumatoid factor negative patients respectively.

Further adjustment for nonbiologic DMARD use in the analyses in Tables 2–4 did not materially alter the results (data not shown).

### 3.4. IL-6, Resistin, and Endothelial Activation

After adjustment for age, sex, and ethnic grouping, IL-6 concentrations were associated with those of resistin (partial *R* = 0.204 (*P* = 0.002)). In a further model, both resistin concentrations and those of IL-6 were related to the endothelial activation score independent of demographic characteristics as well as one another (partial *R* = 0.174 (*P* = 0.01) and partial *R* = 0.395 (*P* < 0.0001)).

## 4. Discussion

This study shows that conventional and non-conventional cardiovascular risk factors associate with circulating concentrations of molecules that mediate endothelial cell activation, the initial stage of atherosclerosis [1–4] in RA. In addition, IL-6 concentrations were found to be most markedly related to this process of early atherogenesis. Interestingly, this relationship was further noted regardless of race (both in black and white patients), sex, advanced age, classic cardiovascular risk factors (namely, obesity, hypertension, and a high atherogenic ratio), and prolonged disease duration and RA severity (moderate or high disease activity and more marked joint damage).

Increased cytokine production is strongly implicated in the documented enhanced cardiovascular disease risk in RA [38]. IL-6 is the most abundant cytokine in the circulation [39, 40] and has increasingly recognized crucial proatherogenic properties [3, 41, 42], is released by endothelial cells upon activation, and affects T and B lymphocytes and the production of acute phase proteins including CRP and fibrinogen by the liver [42–45]. IL-6 thereby integrates endothelial cells in immunological circuits and the acute phase response [42]. TNF-*α* and IL-1 are major inflammatory cytokines in RA joints and stimulate synovial fibroblasts to produce IL-6 [9]. Concentrations of IL-6 but not TNF-*α* and IL-1 are reportedly associated with circulating soluble adhesion molecules in RA [9]. IL-6 is also importantly involved in the pathogenesis of RA as evidenced by the large impact of IL-6 blockade on joint inflammation in this disease [23, 24, 46]. IL-6 concentrations predict incident cardiovascular events in the general population [3]. In the PRIME study, CRP, IL-6, and fibrinogen concentrations each predicted future myocardial coronary death independent of traditional risk factors but only IL-6 associated with incident cardiovascular mortality when all 3 inflammatory markers were entered together in a mixed regression model [47]. In the present investigation, in contrast to IL-6 concentrations, those of CRP were not associated with endothelial activation.

We previously reported a relationship of EGFR and IL-6 concentrations with those of adhesion molecules in RA [9]. The main limitation of the latter study was the small cohort size that likely accounted for the limited number of identified associations [9] and, also, concentrations of MCP-1, an essential molecule in early atherosclerosis [3, 4], were not evaluated. In the present investigation, conventional risk factors as well as RA characteristics that increase cardiovascular risk [16, 17] and are routinely available in clinical practice contributed substantially and significantly less than IL-6 concentrations to the variation in overall early atherogenesis as estimated by an SD score of endothelial activation molecule concentrations. Indeed, IL-6 concentrations were not only associated with those of each endothelial activation molecule but additionally explained 18% of the variation in the endothelial activation score beyond other risk factors.

The current recommendations by the European League Against Rheumatism on cardiovascular risk estimation in RA comprise consideration of traditional risk factors as well as the RA characteristics of disease duration, rheumatoid factor, or antibodies to citrullinated peptide and extraarticular manifestations [48]. However, this approach was based on available but nevertheless relatively low quality evidence [49] and indeed may underestimate the actual cardiovascular risk in individual patients [50, 51]. Systemic inflammation biomarker evaluation improves cardiovascular disease risk prediction in non-RA subjects [52, 53]. Importantly also, whereas consistent disparities in the associations of cardiovascular risk factors including CRP concentrations, blood pressure, lipid levels, obesity and metabolic syndrome with cardiovascular disease in black compared to white African patients with RA were recently identified by us [27–29], ethnicity did not impact the IL-6-endothelial activation relationship in the present investigation. Our results suggest that the role of IL-6 concentrations in predicting cardiovascular event rates and cardiovascular risk stratification in RA deserves further investigation.

We found 2 paradoxically inverse relationships between cardiovascular risk factors and endothelial activation. These comprised the associations of waist circumference with VCAM-1 concentrations and LDL cholesterol concentrations with those of ICAM-1 in all and African black patients with RA, respectively. Both positive and inverse relationships between adiposity indices and lipid concentrations and cardiovascular risk were reported in RA [27, 54–58]. Paradoxically inverse relationships between cardiovascular risk factors and disease may reflect confounding by inflammation [54–58] and indicate that the optimal lipid threshold for employing lipid lowering agents may be lower in patients with high-grade compared with those that have low-grade systemic inflammation in RA [56]. In a very recently reported novel mouse model resembling RA and atherosclerosis [59], a lipid paradox, comprised of the presence of reduced cholesterol and HDL cholesterol concentrations and similar to that reported in patients with RA that sustain the greatest inflammatory burden [56, 60], was documented. However, treatment with etanercept, a TNF-*α* inhibitor, improved arthritis and atherosclerosis without affecting lipid concentrations, suggesting a noncausal relationship between dyslipidemia and atherosclerosis [59]. Indeed, the true impact of lipid metabolism on cardiovascular disease in RA may escape identification upon considering only lipid profiles as routinely performed [61, 62]. Thus, chronic inflammation induces the appearance of proinflammatory HDLs and oxidized LDLs and VLDLs that are likely involved in cardiovascular disease amongst patients with chronic inflammatory diseases [61, 62].

The use of glucocorticoids has been reported to be complicated by enhanced atherosclerosis and proinflammatory HDLs [63] as well as cardiovascular event rates [64] in RA. Although only 6 patients in the current study were current prednisone users, this intervention was independently associated with increased endothelial activation and population origin did not impact on this relationship. However, reported evidence indicates that chronic inflammation is probably the most important mechanism associated with the increased cardiovascular mortality observed in RA. Due to this, whether the potential glucocorticoid related improvement in the inflammatory burden associated with the use of these drugs may prevail in individuals with severe disease over their adverse metabolic effects in terms of the cardiovascular outcome of RA remains to be elucidated [65, 66]. Therefore, cohorts that include numbers of patients on glucocorticoids that are larger than those in the present investigation are needed to confirm or disprove their adverse influence on endothelial activation.

We recently reported evidence for a contribution of resistin to the link between systemic inflammation and cardiovascular risk in RA [37]. Our current finding that IL-6 concentrations relate to those of resistin and that IL-6 and resistin concentrations are associated with endothelial activation independent of one another supports our previous study [37] as well as the potential use of both of these biomarkers in cardiovascular risk stratification in RA.

Our study has other limitations. The cross-sectional design of this investigation precludes drawing inferences on the direction of causality. We did not record menopausal status and cumulative prednisone dose that could be important in the present context. As applies to most reported investigations on cardiovascular risk, we evaluated many relationships. Nevertheless, our main findings each originates in multiple confounder adjusted mixed regression models. In this regard, although many potentially confounding or explanatory characteristics were included in our models and some of them in Table 4 may be overfitted, a remarkable feature of our data analysis was the consistency of associations before being compared with after comprehensive adjustment. Also, when covariates were entered one at a time in separate models, IL-6 remained consistently associated with endothelial activation (data not shown). Finally, characteristics that could be of potential interest in the present context including anticyclic citrullinated antibodies, cytokines other than IL-6, shared epitope, endothelial dysfunction and atherosclerosis were either not routinely or not at all assessed for the purpose of the present investigation.

In conclusion, the most novel and striking finding produced by the present study is that IL-6 concentrations independently contribute markedly more than other cardiovascular risk factors to the variation in overall early endothelial activation in patients with RA. Assessment of IL-6 concentrations may enhance cardiovascular risk stratification in RA.

## Figures and Tables

**Table 1:** Clinical characteristics and endothelial activation molecule and resistin concentrations in black and white patients with RA.

Characteristics	Black (*n* = 112)	White (*n* = 105)	*P*
Age, yrs	55.8 (10.2)	58.2 (10.9)	0.1
Female sex	**89.3**	**79.1**	**0.04**
*Conventional cardiovascular risk factors *			
Smoking			
Current	3.6	9.6	0.09
Ever	**50.0**	**12.5**	**<0.0001**
BMI, kg/m^2^	**29.5 (6.6)**	**25.7 (4.7)**	**<0.0001**
Waist circumference, cm	**93.6 (13.5)**	**89.4 (13.5)**	**0.02**
Waist-hip ratio	**0.85 (0.80–89.5)**	**0.87 (0.81–0.93)**	**0.05**
Hypertension	**74.1**	**49.5**	**0.0002**
SBP, mmHg	**140 (25)**	**130 (17)**	**0.0007**
DBP, mmHg	**86 (15)**	**80 (9)**	**0.0001**
Chol, mmol/L	**4.6 (0.9)**	**5.0 (1.1)**	**0.005**
HDL chol, mmol/L	1.50 (1.30–1.80)	1.59 (1.30–1.99)	0.09
LDL chol, mmol/L	**2.5 (0.8)**	**2.8 (0.9)**	**0.02**
Triglycerides, mmol/L	1.0 (0.7–1.39)	1.0 (0.8–1.4)	0.7
Non-HDL chol, mmol/L	3.1 (0.9)	3.3 (1.0)	0.07
Chol-HDL chol ratio	3.2 (1.0)	3.2 (1.0)	0.9
Chol-HDL chol ratio > 4	20.2	16.5	0.5
Diabetes	**17.0**	**7.6**	**0.04**
Glucose, mmol/L	**4.9 (4.5–5.4)**	**4.7 (4.4–5.0)**	**0.03**
EGFR, mL/min	**104 (91–126)**	**89 (75–103)**	**<0.0001**
Cardiovascular drugs			
Antihypertensive agents	55.4	43.8	0.09
Statins	**20.5**	**37.1**	**0.007**
Ezetimibe	0.0	13.4	…
Oral glucose lowering agents	**35.2**	**21.4**	**0.02**
Insulin	0.9	1.9	0.5
RA characteristics			
Disease duration, yrs	13.0 (9.3)	14.5 (9.4)	0.3
Rheumatoid factor positive	76.8	78.9	0.8
Deformed joints, *n*	**88 (3–15) **	**4 (0–17)**	**0.04**
DAS28	4.2 (1.3)	3.7 (1.6)	0.8
ESR, mm/hr	**21 (9–39)**	**7 (3–15)**	**<0.0001**
CRP, mg/L	**7.0 (4.0–15.0) **	**4.0 (1.5–12.1)**	**0.003**
IL-6, pg/mL	**3.9 (2.5–6.3)**	**3.2 (2.0–5.8)**	**0.003**
Prednisone use	1.8	2.9	0.6
Endothelial activation molecules			
MCP-1, pg/mL	**349.32 (224.76–665.07)**	**472.01 (331.91–683.47)**	**0.006**
VCAM-1, ng/mL	841.84 (696.33–1071.75)	822.09 (638.55–1028.57)	0.4
ICAM-1, ng/mL	**238.10 (170.93–314.52)**	**309.19 (252.09–383.97)**	**<0.0001**
E-selectin, ng/mL	**42.23 (19.78)**	**35.51 (16.53)**	**0.7**
Resistin, ng/mL	37.82 (24.00–56.32)	32.00 (21.62–50.30)	0.2

Dichotomous variables are expressed as proportions or percentages and continuous characteristics as mean (SD) or median (interquartile range). Significant differences are shown in bold. Yrs: years; BMI: body mass index; SBP: systolic blood pressure; DBP: Diastolic blood pressure; chol: cholesterol; HDL: high-density lipoprotein; LDL: low-density lipoprotein, EGFR: estimated glomerular filtration rate; *N*: Number of; DAS28: disease activity score in 28 joints; ESR: erythrocyte sedimentation rate; CRP: C-reactive protein; IL-6: interleukin-6; MCP-1: monocyte chemoattractant protein-1; VCAM-1: vascular adhesion molecule-1; ICAM-1: intercellular adhesion molecule-1. DAS28 reflects moderately active disease in both black and white patients.

**Table 2:** Independent relationships of modifiable conventional cardiovascular risk factors and disease characteristics with endothelial activation in 217 patients with RA.

Characteristics	MCP-1*	VCAM-1*	ICAM-1*	E-selectin	Endothelial activation score
Ever smoking	0.105	−0.091	0.137**	−0.004	0.062
Waist circumference	0.104	−0.166^†^	−0.077	0.063	−0.076
Hypertension	0.056	0.006	−0.113	0.169^†^	0.051
DBP	0.078	0.029	0.040	0.145^†^	0.102
EGFR*	−0.131	−0.109	−0.153	−0.090	−0.205^§^
Deformed joints*	0.013	0.174^†^	−0.073	−0.036	0.034
ESR*	0.012	0.111	0.025	0.204^†^	0.159
IL-6*	0.330^§^	0.246^§^	0.168^†^	0.250^§^	0.423^§^
Prednisone use	0.134	0.061	0.071	0.123	0.165^†^

Results are expressed as partial correlation coefficient as obtained in multivariable models in which age, sex, and cardiovascular risk factors including conventional risk factors (body mass index, hypertension, dyslipidemia (cholesterol-HDL cholesterol ratio > 4 and use of lipid lowering therapy), smoking, and diabetes) and RA characteristics (disease severity (deformed joints), disease activity score in 28 joints, and prednisone use) were adjusted. Significant relationships are shown in bold type. MCP-1: monocyte chemoattractant protein-1; VCAM-1: vascular adhesion molecule-1; ICAM-1: intercellular adhesion molecule-1; DBP: diastolic blood pressure; EGFR: estimated glomerular filtration rate; ESR: erythrocyte sedimentation rate; IL-6: interleukin-6; LDL: low-density lipoprotein; *logarithmically transformed, ^∗∗,†,§^significance at 0.05, <0.05 to 0.01, and <0.01 to <0.0001, respectively.

**Table 3:** Independent associations between cardiovascular risk factors and the endothelial score when entered into the same model.

Characteristics	Partial *R*	*P*
eGFR*	0.203	0.005
ESR*	0.164	0.03
IL-6*	0.392	<0.0001
Prednisone use	0.187	0.01

Model *R* ^2^	0.272	

Risk factors (except for diastolic blood pressure and disease duration because of collinearity with hypertension and number of deformed joints, resp.) that were associated with endothelial activation in previous analyses together with age, sex, and other cardiovascular risk factors (see footnote of Table 2) were entered into the model. Only results on those risk factors that remained independently associated (*P* ≤ 0.05) with the endothelial activation score are shown. EGFR: estimated glomerular filtration rate; ESR: erythrocyte sedimentation rate; IL-6: interleukin-6. *Logarithmically transformed.

**Table 4:** Independent associations between interleukin-6 concentrations and endothelial activation score in subgroups.

Subgroups	Partial *R*	*P*
Population grouping		
Black (*n* = 112)	0.416	<0.0001
White (*n* = 105)	0.378	0.0002
Age		
≤55, y (*n* = 90)	0.366	0.002
>55, y (*n* = 127)	0.415	<0.0001
Sex		
Women (*n* = 183)	0.382	<0.0001
Men (*n* = 34)	0.418	0.05
Smoking		
Never (*n* = 150)	0.349	<0.0001
Ever (*n* = 66)	0.441	0.001
Missing data (*n* = 1)		
BMI, kg/m^2^		
≤29.9 (*n* = 147)	0.354	<0.0001
>29.9 (*n* = 65)	0.506	0.0005
Missing data (*n* = 5)		
Hypertension		
No (*n* = 82)	0.265	0.03
Yes (*n* = 135)	0.452	<0.0001
Chol-HDL chol ratio > 4		
No (*n* = 173)	0.365	<0.0001
Yes (*n* = 39)	0.422	0.03
Missing data (*n* = 5)		
RA duration, y		
≤10 (*n* = 100)	0.327	0.003
>10 (*n* = 116)	0.437	<0.0001
Missing data (*n* = 1)		
Rheumatoid factor		
Negative (*n* = 48)	0.311	0.08
Positive (*n* = 168)	0.406	<0.0001
Missing data (*n* = 1)		
DAS28		
≤2.6 (*n* = 41)	0.268	0.05
>2.6 (*n* = 176)	0.443	<0.0001
Deformed joints		
≤6 (*n* = 123)	0.335	0.002
>6 (*n* = 119)	0.439	<0.0001
Missing data (*n* = 1)		

Age, sex, and cardiovascular risk factors were adjusted in each model as in Table 2. BMI: body mass index; HDL: high-density lipoprotein; RA: rheumatoid arthritis; DAS28: disease activity score in 28 joints (values of ≤2.6 and >2.6 indicate the presence of disease remission or mild disease activity and moderate or high disease activity, resp.).
